# When Gains Go Wrong: A Case of Selective Androgen Receptor Modulator-Related Liver Injury

**DOI:** 10.7759/cureus.87376

**Published:** 2025-07-06

**Authors:** Dhruva Govil, Rana Afram, Amanda Alkhafaji, Everett Woods, Saif Affas

**Affiliations:** 1 Internal Medicine, Henry Ford Providence Hospital, Southfield, USA; 2 Gastroenterology, Henry Ford Providence Hospital, Southfield, USA

**Keywords:** case report, drug-induced liver injury, hepatotoxic drugs, sarm, stenabolic

## Abstract

Selective androgen receptor modulators (SARMs) causing drug-induced liver injury is a rare, albeit inadequately described, potentially serious side effect for those in the fitness industry looking to maximize muscle growth, strength gain, and fat loss as quickly as possible. We present a case of a patient with drug-induced liver injury after starting Stenabolic, a newer SARM.

We report a case of a 40-year-old male who presented with vague gastrointestinal symptoms. Before the presentation, he was relatively healthy but taking multiple over-the-counter supplements. Although he had been taking most of these supplements for a long time without notable side effects, he had recently started taking Stenabolic, a performance-enhancing drug under the SARM category. Laboratory and imaging studies confirmed hepatocellular injury. After ruling out infectious and autoimmune etiology, it was thought that the likely source was Stenabolic. The patient was treated with supportive care and was advised to discontinue Stenabolic. Upon discharge, he began to show clinical improvement.

Although there is limited research about Stenabolic, other agents in the SARM class have been implicated in similar patterns of liver injury. Its structural and pharmacologic similarities to anabolic steroids raise concern for hepatotoxicity through an idiosyncratic immune-mediated mechanism. This case highlights the potential hepatotoxicity of performance-enhancing supplements like Stenabolic. With the growing popularity of SARMs and limited regulation, healthcare providers should maintain a high index of suspicion for supplement-induced liver injury. Further research is needed to clarify the safety and mechanisms of these agents. Until then, their use should be discouraged.

## Introduction

Bodybuilding is an ever-growing industry, with supplements becoming a staple among many fitness enthusiasts despite the absence of FDA regulation. Muscle growth promoters, mainly testosterone, anabolic androgenic steroids (AAS), growth hormones, and selective androgen receptor modulators (SARMs), are some of the key substances that have caught the attention of muscle-building enthusiasts. Although banned in professional sports, the accessibility of purchase through online avenues makes them easier to use. 

Due to concerns of toxicity related to anabolic steroids, SARMs were developed as a nonsteroidal alternative by acting at the cytoplasmic androgen receptor of muscles and bone. Through this mechanism, SARMs were thought to offer tissue-specific benefits without off-target side effects [1]. Despite being developed for potential clinical use in conditions like muscle wasting and osteoporosis, SARMs are often marketed online as performance-enhancing supplements and are frequently misused by individuals outside of medical supervision. However, in 2017, the United States Food and Drug Administration (FDA) issued a warning that SARMs carry an increased risk of cardiovascular and hepatic toxicity [2].

To date, there have been limited reports regarding liver injury due to SARMs. In the following case, we present a patient who developed liver injury due to Stenabolic, a newer SARM, and how it was managed.

## Case presentation

A 40-year-old African American male presented to the hospital with vague gastrointestinal symptoms. Prior to presentation, he was healthy but had been taking multiple over-the-counter supplements. Although he had been using most of these supplements for a long time without notable side effects, he had recently begun taking Stenabolic, a performance-enhancing drug under the SARM category. His clinical presentation included scleral icterus, jaundice, right upper quadrant pain on deep palpation, and dark urine.

Further workup revealed a hepatocellular pattern of liver injury, with elevated total bilirubin, direct bilirubin, and aspartate transaminase/alanine aminotransferase (AST/ALT). His alkaline phosphatase and partial thromboplastin time/prothrombin time/international normalized ratio (PTT/PT/INR) were normal (Table 1).

**Table 1:** Lab Value Progression AST: aspartate transaminase, ALT: alanine aminotransferase, PTT: partial thromboplastin time, PT: prothrombin time, INR: international normalized ratio.

Lab Value	Reference Range (Normal Range)	Admission	Day 2	Day 3	Day 4	Day 5
Bilirubin total (mg/dL)	0.1-1.2	7.7	6.7	6.6	6.8	7.0
Bilirubin direct (mg/dL)	0.0-0.3	5.3	-	-	4.6	-
Alkaline phosphatase (unit/L)	20-120	101	89	95	101	113
AST (unit/L)	10-40	64	54	59	53	47
ALT (unit/L)	10-40	108	91	92	86	77
PT (seconds)	10.0-13.5	11.3	-	-	11.1	-
PTT (seconds)	25.7-37.5	29.6	-	-	27.8	-
INR	0-1.1	0.5	-	-	0.7	-

**Figure 1:**
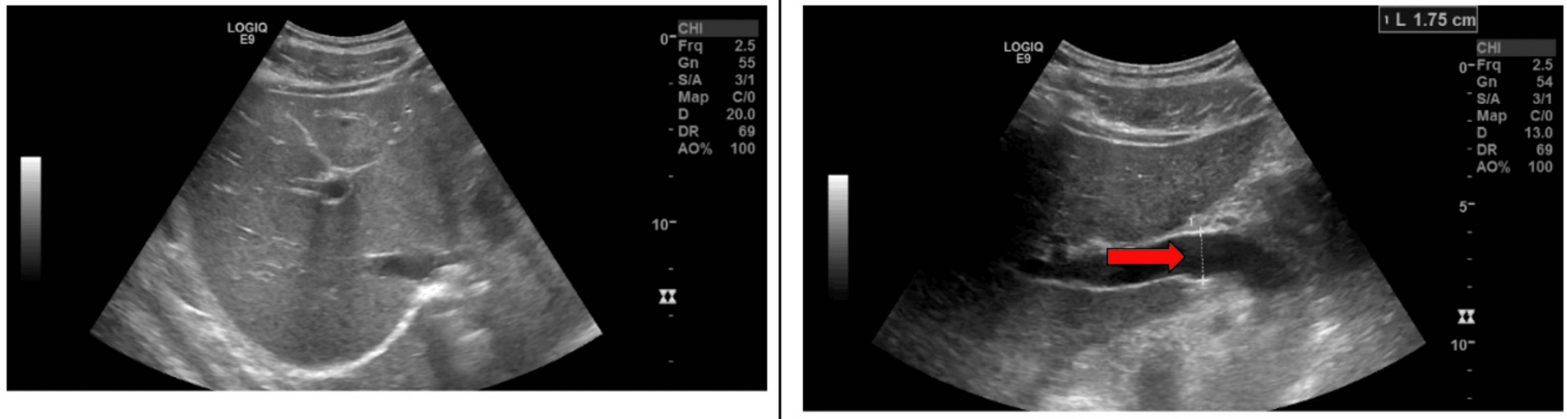
Hepatic Ultrasound (US) Left: US reveals heterogeneous hepatic architecture, no mass, normal gallbladder, and normal common bile duct. Right: US reveals mild dilation of the portal vein at 1.5 cm. No portal vein thrombosis seen.

A right upper quadrant (RUQ) ultrasound revealed heterogeneous hepatic architecture, which was otherwise unremarkable except for a mildly dilated portal vein measuring 1.5 cm. Liver Doppler ruled out portal vein thrombosis (Figure 1). A focused medical history ruled out other potential causes of liver injury. Viral hepatitis panels (including hepatitis A, B, and C) and autoimmune markers were all negative. The patient denied use of alcohol, acetaminophen, or other known hepatotoxins and had no history of chronic liver disease. He received supportive care and was advised to discontinue SARM usage. Upon discharge, his liver enzymes had begun down-trending, with clinical improvement.

## Discussion

Although there is minimal research regarding liver injury due to Stenabolic, similar drugs within the same class have been known to cause the same type of injury. Two case reports described the use of notable SARMs, Ligandrol and Ostarine, in two young males who developed drug-induced liver injury. Interestingly, their clinical presentations were almost identical to our case [1,3]. Similar to our case, Koller et al. reported that these two males presented with symptoms of hepatic dysfunction, elevated AST, ALT, and total bilirubin with conjugated bilirubin predominance, along with normal alkaline phosphatase. Treatment of these patients consisted of discontinuing SARMs, along with deoxycholic acid for two months for the first patient and deoxycholic acid, N-acetylcysteine, and milk thistle for the second patient. These patients’ symptoms and laboratory abnormalities resolved without any further sequelae [3].

Broader case reports examining SARMs, such as Ligandrol, Ostarine, and Testolone, have shown cholestatic or mixed liver injury in otherwise healthy young men. As with our case, they had similar findings of hyperbilirubinemia, transaminitis, and modestly elevated alkaline phosphatase levels. Furthermore, liver biopsies in these cases demonstrated bland canalicular cholestasis with minimal inflammation, a pattern classically associated with anabolic steroid-induced liver injury [4-7]. Specifically, two different cases involving Testolone showed cholestatic hepatitis with high bilirubin, modest transaminase elevations, and histologic findings of lobular cholestasis and ceroid-laden Kupffer cells [4,5]. The similarity across these reports supports a class-wide hepatic risk profile among SARMs, including compounds like Stenabolic.

The physiologic mechanisms of SARM-induced liver injury are still unknown. However, several theorized mechanisms exist. The similarities between anabolic steroids and SARMs could likely play a role via an idiosyncratic immune response [3]. Another possible mechanism is the metabolism and creation of a myriad of metabolites, mainly the N-dealkylated metabolite M1, within the liver, which are susceptible to haptenization and secondary immune response [8]. An additional mechanism is the possibility that SARMs impair bile acid transportation at the canalicular membrane, leading to intrahepatic cholestasis, bile stasis, and hepatocyte injury. This mechanism, seen in some cases, appeared idiosyncratic and dose-independent, consistent with delayed symptom onset and prolonged resolution even after drug cessation [7].

While Stenabolic is commonly categorized as a SARM, it technically functions as a Rev-ErbA ligand and has not been studied in human clinical trials. It has, however, been studied in animals. Those studies showed a potential effect on mitochondrial metabolism and circadian regulation [9]. Due to the lack of purity regulation, it is difficult to determine whether the extent of injury was caused by Stenabolic or other contaminant SARMs. Nevertheless, our patient’s clinical presentation mirrors the hepatic injury patterns reported with other SARMs. To further assess causality, we applied the Roussel Uclaf Causality Assessment Method (RUCAM), which yielded a score in the “probable” range, suggesting a likely association between Stenabolic use and liver injury.

As seen with prior cases, our patient exhibited the hallmark features of SARM-induced liver injury: delayed symptom onset after supplement use, hepatocellular/cholestatic injury, and gradual resolution after discontinuation. While a liver biopsy was not performed, the clinical findings were consistent with those in previous cases. To our knowledge, this is the first published case suggesting a potential link between Stenabolic and drug-induced liver injury in a human subject.

Due to the limited research on Stenabolic and SARMs in general, there is no definitive answer regarding whether this was the cause of the liver injury. Further research is needed to determine whether SARM utilization is beneficial or harmful.

## Conclusions

Stenabolic is a SARM with potential hepatotoxicity. Due to the limited research behind this product, further investigation is needed regarding Stenabolic and SARMs in general. Until then, consumers should be advised against using this product, particularly given the risks associated with unregulated supplement use. This case underscores the importance of clinician awareness when evaluating patients with unexplained liver injury, especially in those who use performance-enhancing supplements. Additionally, increased regulatory oversight and clearer public health guidance are needed to address the growing accessibility and misuse of SARMs.
